# Antioxidative and Anti-Inflammatory Protective Effects of *β*-Caryophyllene against Amikacin-Induced Nephrotoxicity in Rat by Regulating the Nrf2/AMPK/AKT and NF-*κ*B/TGF-*β*/KIM-1 Molecular Pathways

**DOI:** 10.1155/2022/4212331

**Published:** 2022-08-26

**Authors:** Bodour S. Rajab, Talat A. Albukhari, Anmar A. Khan, Bassem Refaat, Samah J. Almehmadi, Nani Nasreldin, Gehad E. Elshopakey, Mohamed El-Boshy

**Affiliations:** ^1^Laboratory Medicine Department, Faculty of Applied Medical Sciences, Umm Al-Qura University, Al Abdeyah, PO Box 7607, Makkah, Saudi Arabia; ^2^Department of Immunology and Hematology, Faculty of Medicine, Umm Al-Qura University, Makkah, Saudi Arabia; ^3^Department of Pathology and Clinical Pathology, Faculty of Veterinary Medicine, New Valley University, El-Kharga, P.O. Box 72511, Egypt; ^4^Department of Clinical Pathology, Faculty of Veterinary Medicine, Mansoura University, Mansoura, Egypt

## Abstract

Herein, the molecular pathogenic pathways implicated in renal injury triggered by amikacin (AK), together with the alleviating actions of *β*-caryophyllene (BCP), were investigated. Adult male Wistar rats (*n* = 32) were disseminated to the four following groups (*n* = 8/group): normal group, positive control animals (PC) that received AK intraperitoneal injections for 14 days (500 mg/kg/day), and rats that received AK simultaneously with small (200 mg/kg/day) and high doses (400 mg/kg/day) of BCP. The PC renal tissues revealed abnormal histology alongside increased apoptosis and significantly elevated serum creatinine and urea with marked proteinuria and oliguria relative to the normal rats. Moreover, renal tissues from the PC animals also showed substantial upregulations in NF-*κ*B/TGF-*β*/KIM-1, whilst Nrf2/AMPK/AKT/PCNA declined, at the gene and protein levels in comparison to the normal rats. Additionally, the levels of markers of oxidative stress (MDA/H_2_O_2_/protein adducts) and inflammation (TNF-*α*/IL-1*β*/IL-6/IL-18/TLR/HSP25) were substantially higher in the PC renal specimens, whereas the antioxidants (GSH/GPx/SOD1/CAT) and interleukin-10 decreased, relative to the NC group. Both BCP protocols improved the biochemical markers of renal functions, alleviated renal histopathology and apoptosis, and decreased NF-*κ*B/TGF-*β*/KIM-1 alongside the concentrations of oxidative stress and proinflammatory markers, whilst promoting Nrf2/AMPK/AKT/IL-10/PCNA and the targeted antioxidants. However, the improving effects in the high-dose regimen were markedly stronger than those observed in animals treated with low dose of BCP. In conclusion, the present report is the first to connect NF-*κ*B/TGF-*β*/KIM-1 proinflammatory and Nrf2/AMPK/AKT antioxidative stress pathways with the pathogenesis of AK-induced nephrotoxicity. Additionally, the current report is the first to disclose alleviating activities for BCP against AK-triggered nephrotoxicity by modulating multiple antioxidative stress with anti-inflammatory molecular pathways.

## 1. Introduction

Amikacin (AK) is an aminoglycoside antibiotic frequently utilized for treating infections and neonatal renal abscess caused by gram-negative bacteria [1–4]. Renal tubular cells excrete the drug in urine without metabolism, and nephrotoxicity could occur by excess accumulation of AK in the renal cortex [5]. Suggested mechanisms for AK-induced renal injury include mitochondrial membrane damage and lysosomal dysfunction that subsequently intensify the production of free radicals, thus predisposing to cellular oxidative stress [3, 4]. Although the nephrotoxic effects of AK are dependent on dose and duration of treatment and could vary between individuals, acute renal failure is the most common complication [1, 5].

Although the molecular pathways involved in AK-induced nephrotoxicity are currently unclear, several experimental reports have revealed that AK triggers renal injury by oxidative stress and proinflammatory processes [6–8]. In this respect, many studies have proclaimed that AK accrues in renal tissues, which promotes mitochondrial dysfunction together with enhanced production of free radicals and declines in antioxidant molecules in renal tissues [7, 9]. Persistent oxidative stress subsequently triggers inflammation of renal tissues by upregulating nuclear factor-kappa B (NF-*κ*B), Toll-like receptor (TLR), tumor necrosis factor- (TNF-) *α*, heat shock protein (HSP)25, interleukin- (IL-) 1, IL-6, and transforming growth factor- (TGF-) *β* [10–12]. Chronic oxidative stress and inflammation then incite renal cell damage, promote cell apoptosis, and increase the specific maker of renal tubular damage, kidney injury molecule-1 (KIM-1) [10–12]. In contrast, nuclear factor erythroid 2-related factor 2 (Nrf2) is a vital antioxidant and anti-inflammatory molecule, which is stimulated by protein kinase B (AKT) and mitogen-activated protein kinase (AMPK) [13–15]. Nonetheless, reports related to the roles of NF-*κ*B/TGF-*β*/KIM-1, as well as Nrf2/AKT/AMPK in AK-induced renal injury, are lacking.

Spontaneous recovery from AK-induced renal dysfunction is improbable; also, there is no standard medication against this common complication [16]. Therefore, many experimental studies have suggested that natural antioxidant and anti-inflammatory products could provide an alternative prophylactic and/or therapeutic approaches against AK-induced nephropathy [4, 6, 17, 18]. Under this theme, *β*-caryophyllene (BCP) is a volatile ingredient of numerous plants that is mostly applied for food seasoning [19]. Despite that BCP showed effective antioxidative and anti-inflammatory nephroprotective activities [15, 20, 21], none of the prior studies explored its possible ameliorating actions against AK-induced renal impairment.

Hence, the current report was performed to study the functions of NF-*κ*B/TGF-*β*/KIM-1 with Nrf2/AKT/AMPK pathways in the pathomechanisms of AK-induced renal dysfunction. Additionally, the present study also measured the potential nephroprotective activities of BCP against AK-induced renal injury. A better understanding about the roles of Nrf2/AKT/AMPK and NF-*κ*B/TGF-*β*/KIM-1 molecular pathways in renal impairment associated with AK, as well as the potential effects of BCP on their expression in renal tissues, could provide a better prophylactic strategy against nephrotoxicity induced by aminoglycoside antibiotics.

## 2. Materials and Methods

### 2.1. Study Design and Prophylactic Protocols

Thirty-two male adult (12 weeks of age) Wistar rats (180-200 g/each) were maintained in a controlled environment (24 ± 1°C with 12 h light/dark cycle) with normal chow and unrestricted access to water. The rats were divided after acclimatization to four equal groups, as follows (*n* = 8 rats/group): normal controls (NC), positive controls (PC) that were injected with amikacin only, and the last groups which concurrently received amikacin with either low (200 mg/kg; LD group) or high (400 mg/kg; HD group) oral BCP (Sigma-Aldrich Chemical Co.; MO, USA) doses. The normal group was injected intraperitoneally with normal saline, whilst freshly prepared intraperitoneal amikacin injections (500 mg/kg; AMIKACIN®; EIPICO Pharmaceuticals Co.; 10^th^ of Ramadan City, Egypt) alone or together with low or high oral BCP doses were given once daily to the assigned animals for 14 successive days, as described earlier [11, 15, 22]. Ethical approval was granted by the Ethics Board at Umm Al-Qura University, KSA (AMSEC 17/06-12-21).

### 2.2. Types of Samples

Following collection of 24 h urine samples (24 h-U) by housing each rat in a metabolic cage (Braintree Scientific Inc., MA, USA), euthanasia was done by cervical dislocation on the fifteenth day, as described earlier [23]. Venous blood was collected, and obtained serum was stored till used. Following excision of kidneys, a piece was used for histopathology. Renal total protein was collected by homogenizing a tissue specimen in a radioimmunoprecipitation assay buffer. Protease inhibitors were then added to inhibit protein degradation. The concentrations of total protein were quantified by a bicinchoninic acid assay (Thermo Fisher Scientific; CA, USA). Each sample was then diluted (1000 *μ*g/mL) by deionized water. RNA extraction and cDNA production were achieved by commercial kits (Thermo Fisher Scientific).

### 2.3. Biochemical Markers of Renal Functions

Systemic concentrations of creatinine (Cr), urea, total protein, and albumin with 24 h-U Cr and total protein concentrations were measured on a fully automated Cobas analyzer (Wiener Lab, Switzerland) using a commercially available kit according to the instructor's pamphlet.

The 24 h urine flow and Cr clearance (Cr-Cl) were calculated, as reported earlier [24]:

(1) $$\begin{array}{c} \text{Urine flow }\left(\mu \text{L}/\min\right)=\frac{24\text{ hr urine volume }\left(\text{mL}\right)}{60\;\min\times 24\text{h}=1440}\times 1000,\text{Cr}-\text{Cl }\left(\text{mL}/\min\right)=\frac{Urine\;Cr\;\left(mg/dL\right)\times Urine\;flow\;\left(mL/min\right)}{Serum\;Cr\;\left(mg/dL\right)}. \end{array}$$

### 2.4. Relative Gene Expression

The expression of the targeted genes was measured on a real-time apparatus (QuantStudio™ 3 Real-Time PCR) and by processing each renal cDNA sample in triplicate wells/gene followed by 40 PCR amplification cycles, as described earlier [25]. Each well contained 1 *μ*L of cDNA (25 ng), 2 *μ*L of nuclease-free water, SYBR Green master mix (5 *μ*L), and a mixture of 5 pmol forward and reverse primers (2 *μ*L; Supplementary Table 1). Two negative controls were used as follows: cDNA samples were substituted with nuclease-free water, whilst the other control was replaced with extracted total RNA without reverse transcription. *GAPDH* gene was used for normalization, and the 2^-∆∆Ct^ approach was employed to measure the expression of the genes of interest relative to the *GAPDH* gene.

### 2.5. Immunohistochemistry (IHC)

Protein expression of NF-*κ*B p50, TGF-*β*1, AMPK*α*, AKT1, and Nrf2 was detected with rabbit primary polyclonal IgG antibodies, whereas primary goat polyclonal IgG antibodies were utilized for detecting KIM-1, in renal tissues (Thermo Fisher Scientific). Following overnight incubation of renal tissue sections with the primary antibodies (1 : 200 concentration; 4°C), the slides were washed and incubated for 30 minutes with ImmPRESS® HRP-conjugated Horse Anti-Rabbit or Anti-Goat IgG Plus Polymer Peroxidase Kits (Vector Laboratories Inc.; CA, USA). Rabbit or goat IgG isotype antibodies (Santa Cruz Biotechnology Inc.; TX, USA) were used in replacement of the primary antibodies to control for nonspecific binding. The slides were then observed with a brightfield microscope (Leica Microsystems, Wetzlar, Germany). Images were then acquired from 10 nonintersecting areas/section. The IHC scores were determined by ImageJ software (https://imagej.nih.gov/ij/), as reported earlier [26].

### 2.6. Terminal Deoxynucleotidyl Transferase-dUTP Nick End Labelling (TUNEL) Technique

Detection of DNA damage/apoptosis in renal tissues was done by a Click-iT™ TUNEL Alexa Fluor™ 488 Imaging Assay (Thermo Fisher Scientific). Next, dual expression of proliferating cell nuclear antigen (PCNA) with apoptotic bodies was done by adding anti-PCNA mouse monoclonal IgG antibodies (1 : 100 dilution) for 3 h. Following washing, Alexa Fluor™ 555-conjugated secondary donkey antimouse IgG antibodies were used. The sections were counterstained with DAPI (Thermo Fisher Scientific). All slides were then examined by a fluorescent microscope (Leica Microsystems). Percentages of apoptosis were determined by calculating the apoptotic/necrotic cells in 15 nonintersecting areas/slide, as reported earlier [27].

### 2.7. Enzyme-Linked Immunosorbent Assay (ELISA)

The concentrations of TNF-*α*, IL-1*β*, IL-6, IL-10, IL-18, HSP25, and TLR4 in renal tissue homogenates were measured by ELISA (Cloud-Clone Corp.; TX, USA). Similarly, renal tissue concentrations of malondialdehyde (MDA), protein carbonyl groups, hydrogen peroxide, glutathione (GSH), superoxide dismutase-1 (SOD1), catalase (CAT), and glutathione peroxidase-1 (GPx1) were also measured by ELISA kits (Cell Biolabs, Inc.; CA, USA). The samples were processed by an automated ELISA machine (Poweam Medical Systems Co., Ltd; China).

### 2.8. Statistical Analysis

SPSS software version 25 was used, and data normality and homogeneity were determined by the Kolmogorov and Smirnov test and the Levene test, correspondingly. According to variance equality, one-way ANOVA test with Tukey's HSD or Games-Howell post hoc tests were applied for comparing between the groups. *P* value < 0.05 indicated statistical significance.

## 3. Results

### 3.1. Serum and Urine Markers of Renal Function

Levels of 24 h urine total protein in addition to Cr and urea in serum were significantly higher, whilst serum total protein and albumin together with 24 h urine volume and flow, Cr levels, and Cr clearance declined markedly, in the PC rats relative to the normal animals (Table 1; *P* < 0.01). Both BCP treatment protocols showed significant reductions in serum creatinine and urea with concurrent increases in systemic total protein and albumin levels in addition to 24 h urine amounts and Cr clearance, compared with the PC animals (*P* < 0.05). Nevertheless, the improvements were significantly more pronounced with the high-dose regimen compared to the LD regimen (Table 1; *P* < 0.01). Moreover, all the tested serum and urine markers of renal function in the HD group were similar to the NC animals, except for urine concentrations of total protein that were substantially higher in the prior group (*P* < 0.01).

### 3.2. Morphology of Renal Tissues

**Figure 1:**
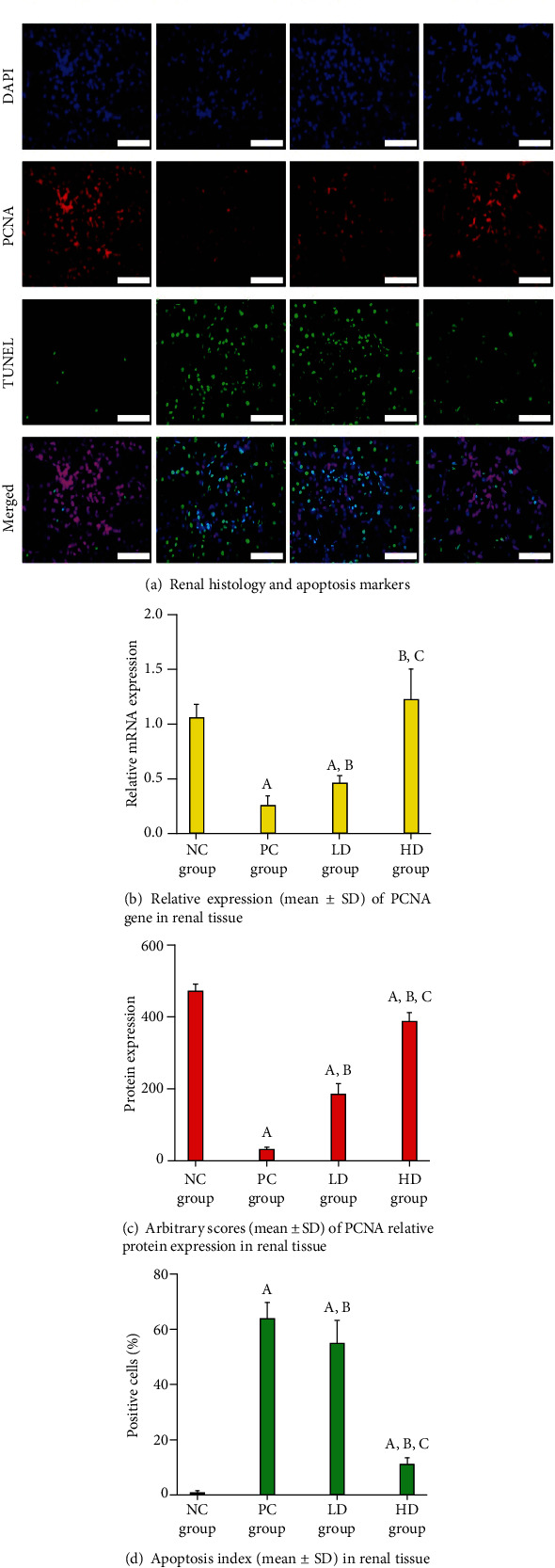
The NC renal tissues stained with H&E showed normal histological characteristics, and the percentages of apoptotic renal cells were scarce, whilst renal expression of PCNA protein was abundant. The PC group displayed substantial glomerular and tubular damage depicted by glomerular basal membrane detachment, with marked leukocytic infiltration. Both HD and LD groups displayed upregulated PCNA mRNA and protein and reduced apoptosis index, significantly relative the PC group.

The NC renal tissues stained with H&E showed normal histological characteristics, and the percentages of apoptotic bodies were scarce, whilst renal expression of PCNA protein was abundant (Figure 1(a)). In contrast, the PC animals revealed substantial glomerular and tubular damage depicted by glomerular basal membrane detachment, cupping of Bowman's capsule, marked protrusion of tubular cells with lumen widening, and interstitial necrosis with marked leukocytic infiltration (Figure 1(a)). Moreover, the mRNA and protein expression of PCNA decreased (*P* < 0.01 for both), whereas apoptosis index increased (*P* < 0.01), drastically in renal specimens from the positive controls compared to normal rats (Figures 1(b)–1(d)). Treatments with both BCP doses in amikacin-intoxicated animals attenuated renal histopathology, upregulated PCNA, and lowered the numbers of apoptotic bodies, significantly relative to the positive control animals (Figure 1; *P* < 0.05). Whilst the HD group displayed substantially enhanced histology and markedly higher PCNA protein with lower percentages of apoptotic cells relative to rats treated with a low dose of BCP (P <0.01), all the markers remained significantly abnormal compared to normal renal specimens (Figure 1; *P* < 0.05 for all).

### 3.3. Renal Expression of Pathogenic and Protective Molecules

**Figure 2:**
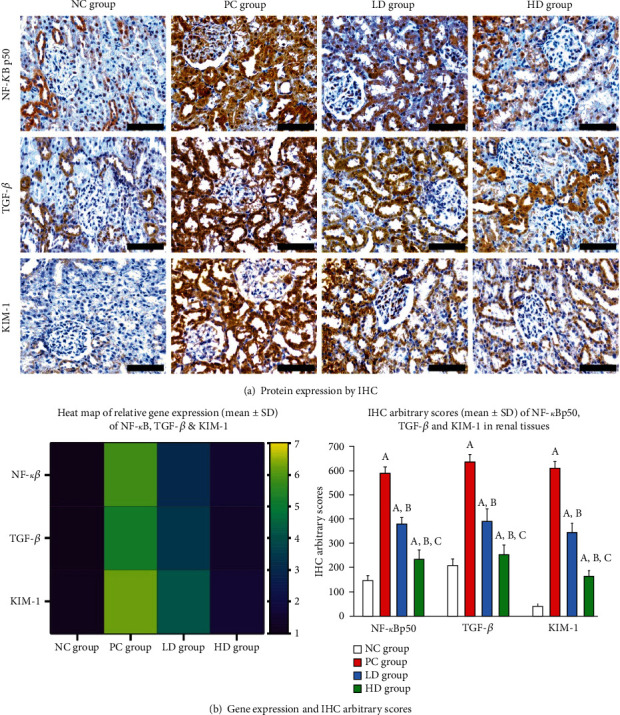
Gene and protein expressions of NF-*κ*B p50, TGF-*β*1, and KIM-1 were detected in the NC renal specimens. The mRNAs and proteins of all molecules increased significantly in the PC renal tissue group. The low and high doses of BCP suppressed the genes and proteins of the molecules of interest compared to those of the PC group.

**Figure 3:**
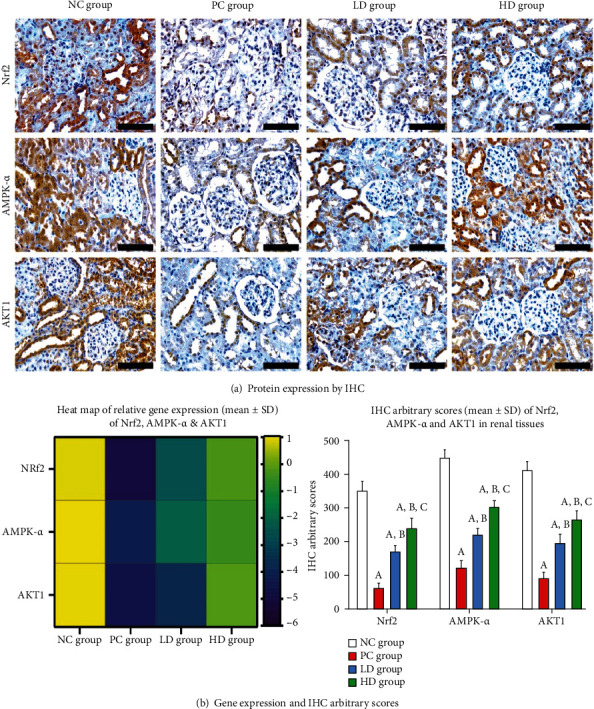
Gene and protein expression of Nrf2, AMPK-*α*, and AKT1 were detected in the NC renal specimens. The mRNAs and proteins of all molecules decreased significantly in the PC renal tissue group. The low and high doses of BCP increased the genes and proteins of the molecules of interest compared to those of the PC group.

NF-*κ*B p50, TGF-*β*1, and KIM-1 (Figure 2) in addition to Nrf2, AMPK-*α*, and AKT1 (Figure 3) were detected at the gene and protein levels in renal tissues from normal rats. Whilst the mRNAs and proteins of NF-*κ*B p50, TGF-*β*1, and KIM-1 increased significantly (Figure 2; *P* < 0.01 for all), those of Nrf2, AMPK-*α*, and AKT1 decreased markedly (Figure 3; *P* < 0.01 for all), in the PC renal samples than in the negative control group. Despite that the low and high BCP doses modulated the expression of the tested molecules relative to the positive control animals, the effects were markedly more manifested in the rats that received a high BCP dose (Figures 2 and 3; *P* < 0.01 for all markers). Nevertheless, the mRNAs and proteins of NF-*κ*B p50, TGF-*β*1, and KIM-1 were substantially higher, whereas those of Nrf2, AMPK-*α*, and AKT1 were substantially lower, in the HD animals relative to the NC renal tissues.

### 3.4. Renal Gene Expression and Concentrations of Inflammatory and Oxidative Stress Molecules

**Figure 4:**
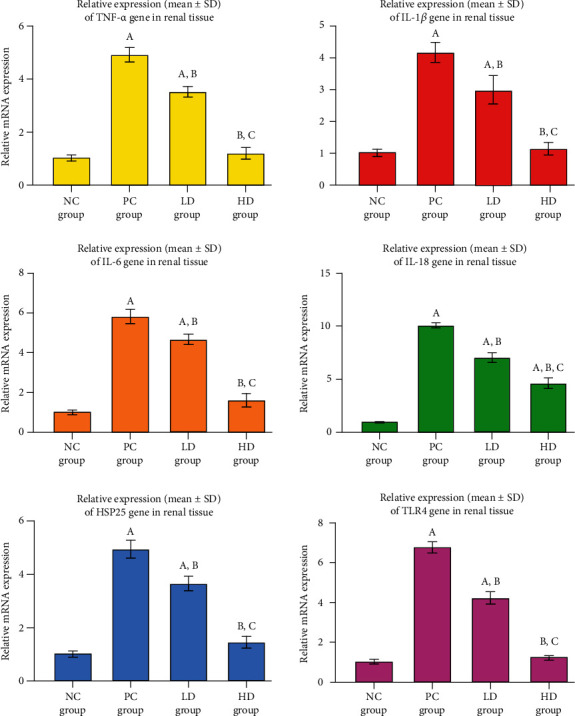
Gene expression of TNF-*α*, IL-1*β*, IL-16, IL-18, HSP25, and TLR4 was detected in the NC renal specimens. The mRNAs of TNF-*α*, IL-1*β*, IL-6, IL-18, HSP25, and TLR4 increased significantly in the PC renal tissue group. The low and high doses of BCP reduced the gene expression of the targeted molecules relative to those of the PC group.

*TNF-α*, *IL-1β*, *IL-6*, *IL-18*, *HSP25*, and *TLR4* mRNAs increased significantly in the renal specimens from the positive controls relative to the normal rats (Figure 3; *P* < 0.01). Treatment with both doses of BCP significantly reduced the mRNAs of *TNF-α*, *IL-1β*, *IL-6*, *IL-18*, *HSP25*, and *TLR4* genes in renal tissues in comparison to the PC rats (Figure 3). The LD group showed drastically higher gene expression of *TNF-α*, *IL-1β*, *IL-6*, *IL-18*, *HSP25*, and *TLR4* in comparison to the negative control animals (*P* < 0.05), whereas the HD and NC groups showed a similar expression profile (Figure 4).

Concurrently, renal concentrations of TNF-*α*, IL-1*β*, IL-6, IL-18, HSP25, TLR4, MDA, H_2_O_2_, and protein carbonyl groups increased, whilst IL-10, GSH, SOD1, GPx1, and CAT decreased, substantially in the PC animals compared to the normal group (Table 2). The concentrations of inflammatory and prooxidative stress markers in renal tissues decreased, whereas those of IL-10 and antioxidants increased, markedly with both BCP regimens relative to the PC animals. Nonetheless, the anti-inflammatory and antioxidant molecules were considerably higher in the high-dose than the low-dose regimen (Table 2). Although the concentrations of TNF-*α*, IL-1*β*, IL-6, TLR4, IL-10, SOD1, GPx1, and CAT were comparable in the HD and NC renal specimens, IL-18, HSP25, MDA, H_2_O_2_, and protein carbonyl groups were markedly higher and concurred with significantly lower GSH and CAT in the animals that received a high dose of BCP (Table 2).

## 4. Discussion

Herein, we explored the molecular pathogenic pathways that could contribute to AK-induced nephropathy. Moreover, we investigated the alleviating actions of BCP against renal oxidative stress and inflammation associated with AK nephrotoxicity. Our results demonstrated significant increases in serum Cr and urea that coincided with recessions in serum concentrations of total protein and albumin together with low urine volume, proteinuria, and reduced urine Cr clearance in the PC rats compared to the normal animals. Renal samples from the PC groups also showed substantial increases in MDA, H_2_O_2_, and protein carbonyl groups alongside declines in renal GSH, SOD1, GPx1, and CAT. Additionally, the proinflammatory markers (TNF-*α*, IL-1*β*, IL-6, IL-18, HSP25, & TLR4) increased, whilst IL-10 decreased, at the gene and protein levels in the PC renal specimens in comparison to the normal rats.

Aminoglycoside antibiotics, including amikacin, are associated with several adverse events, and patients could develop acute renal failure with elevated serum creatinine and urea alongside reductions in urine output and urine creatinine clearance [1, 5]. At the tissue level, AK-induced renal injury is linked with marked glomerular damage, tubular necrosis, interstitial inflammation, and increased leucocytic infiltration [9, 28]. Suggested mechanisms for AK-induced nephropathy include mitochondrial damage with increased ROS production together with declines in renal antioxidant molecules (e.g., GSH/SOD/GPx1/CAT), thus promoting the formation of free radicals, lipid peroxides, and protein adducts that subsequently provoke renal cell damage [3, 4, 7, 9]. Moreover, renal oxidative stress is believed to trigger inflammation by upregulating several proinflammatory cytokines (e.g., IL-1*β*, IL-6, and TNF-*α*, HSP25, and TLR4) with simultaneous inhibition of IL-10, which is a robust anti-inflammatory molecule [10–12]. Our results agree with many prior studies that advocated major roles for oxidative stress and inflammation in the pathomechanisms of renal injury caused by AK [3, 4, 7, 9–12].

Although NF-*κ*B, TGF-*β*, and Nrf2 are chief modulators of redox biology and inflammatory responses in the kidney, little is known regarding their contributions to AK-induced nephrotoxicity. In this regard, several renal pathologies are linked with substantial increases in NF-*κ*B [29, 30] and TGF-*β*1 [31, 32] that consequently stimulate numerous proinflammatory cytokines. Moreover, coactivation of renal NF-*κ*B and TGF-*β* pathways aggravates tissue inflammation and initiates apoptosis [33–35]. Furthermore, both molecules also increased substantially in rat renal tissues with gentamicin treatment, which is another aminoglycoside antibiotic linked with renal injury [36]. On the other hand, Nrf2 is a key molecule that regulates cellular antioxidant pathways, and the protein decreases significantly in renal tissues with a variety of diseases induced by oxidative stress [37, 38]. Additionally, curcumin attenuated gentamycin-induced renal injury by increasing the production of Nrf2 [39]. Similarly, the AMPK/AKT pathway contributes to the regulation of cellular antioxidant and anti-inflammatory networks, and the activation of this pathway alleviated renal injury induced by oxidative stress and proinflammatory stimulants [40–42].

As far as we are aware, this study is the first to reveal drastic elevations in NF-*κ*B, TGF-*β*1, and KIM-1 gene and protein expression, whereas the mRNAs and proteins of AMPK-*α*, AKT1, and Nrf2 decreased, in the renal samples from the PC animals relative to normal rats. Furthermore, the percentages of apoptotic cells increased, whilst the mRNA and protein of PCNA declined, significantly in the positive control renal specimens compared to the NC animals. Therefore, we hypothesize that AK-induced renal cell injury might encompass upregulations of NF-*κ*B and TGF-*β*1 with concurrent downregulations in AMPK-*α*, AKT1, and Nrf2 that trigger cell death via oxidative stress and inflammation with simultaneous inhibition of the survival protein, PCNA [36–40]. Moreover, we suggest that KIM-1 could be utilized to monitor tubular damage during treatment with aminoglycoside antibiotics [15, 43, 44]. However, further work is mandatory to study the connections between the different molecular mechanisms involved in AK-induced nephropathy to corroborate our suggestions.

Prevention and clinical management of AK-induced nephrotoxicity mainly involves using alternative antibiotics, increasing intervals between doses, and inhibition of aminoglycoside reuptake by tubular cells to minimize renal damage [16, 45, 46]. On the other hand, numerous experimental studies have shown renoprotective actions for several antioxidant and anti-inflammatory nutraceutical products against aminoglycoside-induced nephrotoxicity [4, 11, 39, 42]. In the present study, the addition of low or high doses of BCP with AK reduced serum and urine biochemical markers of renal injury, protected against renal histological damage, decreased renal apoptosis index, and increased PCNA gene and protein expression compared to the positive control rats. Additionally, both BCP regimens reduced the renal concentrations of oxidative stress molecules and diminished several proinflammatory cytokines with concomitant increases in the concentrations of antioxidants and IL-10 compared to the renal samples from the PC rats. However, the ameliorating activities were significantly more apparent with the high BCP dose relative to the low-dose group.

BCP is a common natural component of many plants that has shown potent antioxidant and anti-inflammatory actions [47–49]. In this regard, BCP treatment alleviated renal impairment induced by hyperoxaluria in murine by reducing NF-*κ*B and KIM-1, enhancing renal antioxidant mechanisms, and promoting cell survival [50]. Likewise, BCP reduced the concentrations of NF-*κ*B with many inflammatory markers, whilst increasing several Nrf2-dependent antioxidant molecules, hence alleviating the complications associated with high glucose on mesangial cells [51]. Similar effects were also shown after using BCP for attenuating sulfasalazine-induced renal damage that involved reductions in NF-*κ*B and TGF-*β* alongside increased Nrf2, AMPK, and AKT concentrations [15]. BCP therapy also reduced lipopolysaccharide-induced acute lung injury in mice by decreasing a variety of inflammatory cytokines, including TGF-*β* [52]. Likewise, Ullah et al. documented anti-inflammatory actions for BCP by suppressing TLR activity and upregulating the AMPK pathway in the nervous system [53]. BCP also mitigated cerebral injury in rat, as well as inhibited fat deposition in human liver cells, by stimulating the AMPK/AKT pathway [54, 55]. Furthermore, several previous reports have shown that treatment with different doses of BCP for short and long periods of administration was safe and not associated with toxic effects in healthy animals [56, 57].

Our observations correlate with many prior studies and suggest that BCP could be used as a safe prophylactic nephroprotective substance against nephropathy associated with AK treatment by downregulating NF-*κ*B and TGF-*β*1 and simultaneously enhancing antioxidative mechanisms by upregulating Nrf2-regulated molecules in renal tissues [15, 50–52, 54, 55]. However, additional experiments are still required to measure the therapeutic benefit of BCP following inducing renal impairment by aminoglycoside antibiotics.

In conclusion, the current study is the first to demonstrate that renal oxidative stress and inflammation following AK treatment could involve aberrant renal expression of NF-*κ*B, TGF-*β*1, Nrf2, AMPK, and AKT molecular networks. Although low- and high-dose protocols of BCP alleviated AK-induced renal impairment by enhancing antioxidative stress and anti-inflammatory activities, the best ameliorative outcomes were detected with the latter regimen. However, farther studies are essential to measure the therapeutic benefits of BCP against nephrotoxicity and ototoxicity induced by aminoglycoside antibiotics.

## Figures and Tables

**Table 1:** Concentrations (mean ± SD) of serum and 24 h urine markers of renal function.

		NC	PC	LD	HD
Serum	Cr (mg/dL)^#^	0.41 ± 0.08	1.1 ± 0.2^a^	0.74 ± 0.1^a,c^	0.43 ± 0.07^c,e^
	Urea (mg/dL)^$^	34.7 ± 3.6	66.6 ± 6.3^a^	55.4 ± 4.1^a,c^	37.6 ± 4.4^c,e^
	Total protein (g/dL)^$^	7.1 ± 0.4	5.3 ± 0.5^a^	6.2 ± 0.4^a,c^	6.8 ± 0.3^c,d^
	Albumin (g/dL)^$^	4.4 ± 0.4	3.1 ± 0.4^a^	3.6 ± 0.3^a,b^	4.1 ± 0.2^c,d^
24 h urine	Urine volume (mL)^#^	8.8 ± 0.7	3.9 ± 0.3^a^	5.1 ± 0.2^a,c^	8.3 ± 0.4^c,e^
	Urine flow (*μ*L/min)^#^	6.1 ± 0.5	2.7 ± 0.2^a^	3.5 ± 0.2^a,c^	5.8 ± 0.3^c,e^
	Cr (mg/dL)^$^	45.6 ± 5.2	24.3 ± 3.8^a^	30.4 ± 2.6^a,b^	41.7 ± 5.2^c,e^
	Cr clearance (mL/min)^#^	0.7 ± 0.2	0.06 ± 0.02^a^	0.14 ± 0.02^a,c^	0.57 ± 0.15^c,e^
	Total protein (mg/dL)^$^	4.1 ± 0.4	12.1 ± 0.7^a^	7.8 ± 0.5^a,c^	5.3 ± 0.6^a,c,e^

^#^ANOVA with Games-Howell post hoc test. ^$^ANOVA with Tukey's HSD post hoc test. NC: normal group; PC: positive control group; LD: amikacin+low-dose *β*-caryophyllene group; HD: amikacin+high-dose *β*-caryophyllene group. ^a^*P* < 0.01 relative to NC. ^b^*P* < 0.05 relative to PC. ^c^*P* < 0.01 relative to PC. ^d^*P* < 0.05 relative to LD. ^e^*P* < 0.01 relative to LD.

**Table 2:** Concentrations (mean ± SD) of proinflammatory and oxidative stress molecules in renal specimens.

	NC	PC	LD	HD
TNF-*α* (pg/mL)^$^	37.6 ± 6.3	209.4 ± 11.5^b^	77.1 ± 10.7^b,c^	38.4 ± 5.4^c,d^
IL-1*β* (pg/mL)^$^	32.6 ± 6.1	193.6 ± 17.4^b^	101.4 ± 25.3^b,c^	47.5 ± 6.8^c,d^
IL-6 (pg/mL)^$^	66.1 ± 8.4	194.4 ± 21^b^	106.9 ± 9.8^b,c^	62.8 ± 7.6^c,d^
IL-18 (pg/mL)^#^	149.9 ± 16.1	1047.3 ± 81.2^b^	581.1 ± 33.7^b,c^	215.4 ± 20.1^b,c,d^
HSP25 (ng/mL)^#^	66.1 ± 8.4	194.4 ± 21^b^	106.9 ± 9.8^b,c^	62.8 ± 7.6^b,c,d^
TLR4 (ng/mL)^#^	0.88 ± 0.09	3.11 ± 0.20^b^	1.93 ± 0.27^b,c^	1.10 ± 0.23^c,d^
IL-10 (pg/mL)^$^	51.1 ± 7.8	13.8 ± 3.3^b^	27.7 ± 4.7^b,c^	45.2 ± 5.8^c,d^
GSH (mg/g)^$^	46.7 ± 4.5	13.3 ± 3.7^b^	24.1 ± 4.2^b,c^	36.8 ± 4.3^b,c,d^
SOD1 (U/g)^$^	53.7 ± 5.7	19.7 ± 5.6^b^	23.2 ± 5^b^	47.4 ± 6.3^c,d^
GPx1 (*μ*g/mg)^$^	4.7 ± 0.4	1.9 ± 0.4^b^	2.7 ± 0.3^b,c^	4.6 ± 0.5^c,d^
CAT (U/mg)^$^	285.6 ± 16.8	120.2 ± 15.1^b^	166.2 ± 17.6^b,c^	256.4 ± 19.1^a,c,d^
MDA (nmol/g)^$^	29.6 ± 5.1	72.3 ± 5.2^b^	55.4 ± 6.5^b,c^	39.5 ± 5.2^b,c,d^
H_2_O_2_ (*μ*M/g)^∗^^#^	1.09 ± 0.2	66.7 ± 6.3^b^	38.4 ± 4.4^b,c^	2.3 ± 0.7^a,c,d^
Protein carbonyl (nmol/g)^#^	0.5 ± 0.08	5.8 ± 0.5^b^	4.2 ± 0.4^b,c^	1.46 ± 0.21^b,c,d^

^#^ANOVA with Games-Howell post hoc test. ^$^ANOVA with Tukey's HSD post hoc test. TNF-*α*: tumor necrosis factor-*α*; IL-1*β*: interleukin-1*β*; IL-6: interleukin-6; IL-10: interleukin-10; IL-18: interleukin-18; HSP25: heat shock protein 25; TLR4: Toll-like receptor 4; GSH: glutathione; SOD1: superoxide dismutase 1; CAT: catalase; GPx1: glutathione peroxidase-1; MDA: malondialdehyde; H_2_O_2_: hydrogen peroxide; NC: normal group; PC: positive control group; LD: amikacin+low-dose *β*-caryophyllene group; HD: amikacin+high-dose *β*-caryophyllene group. ^a^*P* < 0.05 relative to NC. ^b^*P* < 0.01 relative to NC. ^c^*P* < 0.01 relative to PC. ^d^*P* < 0.01 relative to LD.

## Data Availability

The data used to support the findings of this study may be released upon application to the corresponding author, Mohamed El-Boshy, who can be contacted through the following email: dr_elboshy@yahoo.com.

## Supplementary Materials

Supplementary Materials Supplementary Table 1: the sequences of PCR primers used for the detection of rat GAPDH, Nfkb1, TGF-*β*1, KIM-1 (Havcr1), AMPK-*α*, Nrf2 (Nfe2l2), AKT1, PCNA, TNF-*α*, IL-1*β*, IL-6, IL-18, HSP25 (HSBP1), and TLR4 mRNAs in renal samples including the corresponding gene accession numbers and amplicon sizes.
