# Supplementary material for: Antioxidative and Anti-Inflammatory Protective Effects of β-Caryophyllene against Amikacin-Induced Nephrotoxicity in Rat by Regulating the Nrf2/AMPK/AKT and NF-κB/TGF-β/KIM-1 Molecular Pathways
Source: Oxid Med Cell Longev. 2022 Aug 26;2022:4212331. doi: 10.1155/2022/4212331 (PMC9439917; doi:10.1155/2022/4212331)
Supplement: Supplementary Materials — Supplementary Table 1: the sequences of PCR primers used for the detection of rat GAPDH, Nfkb1, TGF-β1, KIM-1 (Havcr1), AMPK-α, Nrf2 (Nfe2l2), AKT1, PCNA, TNF-α, IL-1β, IL-6, IL-18, HSP25 (HSBP1), and TLR4 mRNAs in renal samples including the corresponding gene accession numbers and amplicon sizes. [file 4212331.f1.pdf]

Supplementary table 1: The sequences of PCR primers used for the detection of rat *GAPDH*, *Nfkb1*, *TGF-β1*, KIM-1 (*Havcr1*), *AMPK-α*, *Nrf2 (Nfe2l2)*, *AKT1*, *PCNA*, *TNF-α*, *IL-1β*, *IL*-6, *IL-18*, *HSP25 (HSBP1), and TLR4* mRNAs in renal samples including the corresponding genes accession numbers and amplicon sizes.

**Genes**	**Forward**	**Reverse**	**Amplicon size**
GAPDH (NCBI: NM_017008.4)	5’ GCA TCT TCT TGT GCA GTG CC 3’	5’ GAG AAG GCA GCC CTG GTA AC 3’	105 bp
Nfkb1 (NCBI: NM_001276711.1)	5’ CTC TCT CGT CCT CCT CCA CA 3’	5’ TTG CGG AAG GAT GTC TCC AC 3’	91 bp
TGF-β1 (NCBI: NM_021578.2)	5’ CCA TGA CAT GAA CCG ACC CT 3’	5’ TGC CGT ACA CAG CAG TTC TT 3’	141 bp
Havcr1 (NCBI: NM_173149.2)	5’ TCA CTG TCC TTC AGG TCA ACA C 3’	5’ GGC CCC AAC ATG TCG TTG T 3’	177 bp
AMPK-α (NCBI: NM_019142.3)	5’ AAG ATC GGC CAC TAC ATC CTG 3’	5’ CAG GTT CTG GAT CTC TCT GCG 3’	171 bp
Nfe2l2 (NCBI: NM_031789.2)	5’ GCA CAT CCA GAC AGA CAC CA 3’	5’ GCT GGG AAT ATC CAG GGC A 3’	157 bp
AKT1 (NCBI: NM_033230.3)	5’ GCC ACG GAT ACC ATG AAC GA 3’	5’ AGT TGT TGA GTG GGG ACT CG 3’	175 bp
PCNA (NCBI: NM_022381.3)	5’ GGG TGA AGT TTT CTG CGA GTG 3’	5’ GCT GAA CTG GCT CAT TCA TCT C 3’	117 bp
TNF-α (NCBI: NM_012675.3)	5’ ATC GGT CCC AAC AAG GAG GA 3’	5’ GGT GGT TTG CTA CGA CGT G 3’	134 bp
IL-1β (NCBI: NM_031512.2)	5’ ACA AAA ATG CCT CGT GCT GTC 3’	5’ CCA CAG GGA TTT TGT CGT TGC 3’	134 bp
IL-6 (NCBI: NM_012589.2)	5’ TCT GGT CTT CTG GAG TTC CGT 3’	5’ TGG AAG TTG GGG TAG GAA GGA 3’	169 bp
IL-18 (NCBI: NM_019165.2)	5’ AGG ACT GGC TGT GAC CCT AT 3’	5’ GTC CTG GCA CAC GTT TCT GA 3’	152 bp
HSBP1 (NCBI: NM_173119.2)	5’ TGG AGT AGA AGA ACT GGA CGC 3’	5’ TGG CTT GGG AAA GTG ATG TTC T 3’	104 bp
TLR4 (NCBI: NM_019178.2)	5’ TGT TCC TTT CCT GCC TGA GAC 3’	5’ AAG ATC TTC AGG GGG TTG AAG C 3’	160 bp
